# Thrombin A-Chain: Activation Remnant or Allosteric Effector?

**DOI:** 10.1155/2010/416167

**Published:** 2010-12-09

**Authors:** Isis S. R. Carter, Amanda L. Vanden Hoek, Edward L. G. Pryzdial, Ross T. A. MacGillivray

**Affiliations:** ^1^Centre for Blood Research, University of British Columbia (UBC), Vancouver, BC, Canada V6T 1Z3; ^2^Department of Biochemistry and Molecular Biology, (UBC), Vancouver, BC, Canada V6T 1Z3; ^3^R and D Canadian Blood Services, Ottawa, ON, Canada K1G 4J5; ^4^Department of Pathology and Laboratory Medicine, (UBC), Vancouver, BC, Canada V6T 1Z3

## Abstract

Although prothrombin is one of the most widely studied enzymes in biology, the role of the thrombin A-chain has been neglected in comparison to the other domains. This paper summarizes the current data on the prothrombin catalytic domain A-chain region and the subsequent thrombin A-chain. Attention is given to biochemical characterization of naturally occurring prothrombin A-chain mutations and alanine scanning mutants in this region. While originally considered to be simply an activation remnant with little physiologic function, the thrombin A-chain is now thought to play a role as an allosteric effector in enzymatic reactions and may also be a structural scaffold to stabilize the protease domain.

## 1. Biosynthesis of Prothrombin

**Figure 1:**
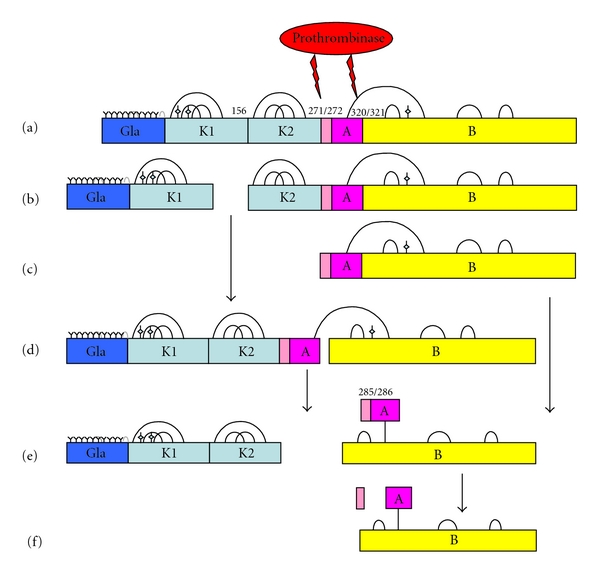
Activation of thrombin by the prothrombinase complex (reviewed in [4]). Prothrombin is colored by domain in this schematic, highlighting the A-chain (pink) and B-chain (yellow). The gamma carboxylated Gla residues are noted by (**Y**) at the N terminus of prothrombin, the carbohydrate attachment sites in kringle 1 (K1) and the B domain are noted by the shaded star, and the disulfide bridges are shown. Factor Xa initially cleaves Prothrombin (a) at Arg 320 to produce meizothrombin (d), followed by cleavage at Arg 271 to release fragment 1.2 from nascent thrombin (e). Thrombin then undergoes intermolecular autolysis to cleave the Arg 285/Thr 286 bond (f), liberating the A13 peptide (pale pink) to generate *α*-thrombin. Experimental constructs used in biochemical studies of the thrombin A-chain include prethrombin-1, which lacks fragment 1 (b) and prethrombin-2 (c).

Prothrombin is a single chain glycoprotein of M_r_ 72,000 that circulates in plasma at a concentration of 100–200 *μ*g/mL. As shown in Figure 1, prothrombin consists of four structural domains: the Gla domain, a region containing 10 *γ*-carboxylated glutamic acid residues which mediate pro thrombin binding to procoagulant phospholipid surfaces; two kringle domains, which are thought to be involved in protein-protein interactions; and lastly the trypsin-like serine protease domain, which contains the enzyme active site. Disulfide bonds and cleavage sites are also shown in Figure 1. Prothrombin also has three N-linked carbohydrate chains at residues Asn78, Asn100 (located in the kringle domain 1), and Asn373 (present in the serine protease domain) [1]. 

Prothrombin is synthesized in the liver as a precursor containing an N-terminal pre-propeptide of 43 amino acids. The presequence, or signal peptide, functions in the cotranslational transfer of the protein across the endoplasmic reticulum membrane and is removed by signal peptidase. The propeptide comprises in part the recognition sequence for a vitamin K-dependent carboxylase that converts the 10 glutamic acid residues in the Gla domain (at the N-terminal region of prothrombin) to *γ*-carboxyglutamic acid or Gla. These modified residues bind calcium (Ca^++^) leading to a conformational change that is essential for assembly of the substrate, prothrombin, with prothrombinase on a procoagulant phospholipid membrane for subsequent activation to thrombin. Following *γ*-carboxylation, the propeptide is removed by enzymatic cleavage C-terminal to an Arg-Arg sequence generating the new N-terminus of the mature zymogen [1]. The final posttranslational modification undergone by prothrombin prior to secretion into the bloodstream is addition of the three N-linked carbohydrate moieties.

## 2. Conversion to Thrombin

When coagulation is initiated at a site of vascular injury, prothrombin is converted to thrombin by prothrombinase (see [2] for a review of the cell-based model of coagulation). As shown in Figure 1, factor Xa cleaves two peptide bonds in prothrombin, initially cleaving at the Arg320-Ile321 bond to generate meizothrombin. This intermediate is then cleaved at the Arg271-Thr272 bond to release fragment 1.2, consisting of the Gla domain and the two kringle domains. Depending on the order of peptide bond cleavage and in the absence of a phospholipid membrane, an alternative intermediate product called prethrombin-2 can also be generated over the course of prothrombin activation [3]. The proteolytically active thrombin molecule is comprised of a 49-amino acid light chain (A-chain) linked by a single disulphide bond to the 259-residue heavy chain (B-chain), which contains three intrachain disulphide bonds. The serine protease domain of thrombin is located in the B-chain. Nascent human thrombin undergoes autoproteolysis at Arg284-Thr285 of the A-chain, releasing a 13-residue N-terminal peptide and yielding a 36-residue A-chain to form *α*-thrombin [4, 5]. It is not currently known whether full A-chain nascent thrombin, truncated A-chain *α*-thrombin, or both forms of thrombin are present when coagulation is initiated *in vivo *or in plasma [1]. 

## 3. Thrombin A-Chain

**Figure 2:**
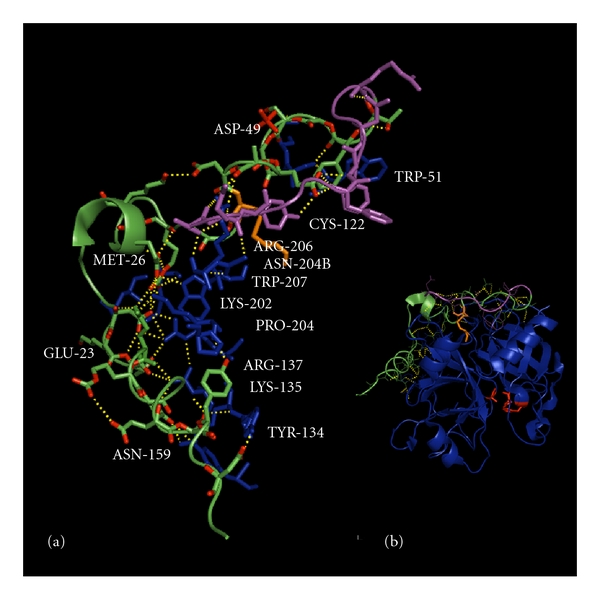
Pymol-generated structure of thrombin using the PDB 3GIS coordinates of Ser195Ala thrombin [8]. (a) Interactions between the A-chain and the B-chain of thrombin (B-chain residues are labeled in blue, the 36-amino acid residue A-chain is colored in green, and the covalently bound A13 peptide segment is colored magenta. The covalent disulfide bridge between the A- and B-chains is through Cys1(93)-Cys122, as shown in orange. Hydrogen bonds are colored yellow and residue numbering is based on chymotrypsinogen, with prothrombin numbering for the A-chain provided in brackets. The A-chain is stabilized by the Asp1a(92)-Lys9(301) and Arg14d(310)-Glu13(314) ion pairs and the ion quartet Arg4(96)-Glu8(300)-Asp14(306)-Glu14c(309). The ionic interactions include Glu8(300)-Lys202-Glu14c(309), Asp14(306)-Arg137, Lys14a(307)-Glu23, and Glu14e(311)-Lys135-Asn159-Tyr194a. Hydrophobic stacking interactions include Tyr14j(316)-Pro204 and Phe1m(280)-Phe1l(281)-Phe1g(286) in the A13 peptide of the A-chain. (b) Thrombin structure showing active site residues in red and location of the A-chain on the opposite face of the molecule.

As shown in Figure 2, the A-chain of thrombin extends along the surface of the B-chain in a shallow curved groove, arranged in a boomerang-like shape opposite the active site [6]. X-ray crystallography structures of human thrombin show that the A-chain hinges the two interacting six-stranded beta barrels of the B-chain. The A-chain interacts with the B-chain through a network of six buried salt bridges and ionic interactions, and ten interchain H-bonds that stabilize the orientation of the B domain barrels. The A-chain is further stabilized through several intramolecular polar and salt bridge interactions [7]. The central portion of the A-chain is most rigid, due to the presence of strong salt bridge interactions (Figure 2). The X-ray crystal structure of thrombin also reveals that both termini of the A-chain are disordered, raising the question: “How much of the A-chain is required to maintain thrombin stability and activity?” The C-terminal segment up to Tyr14j is an amphiphilic alpha-helix forming one and a half turns, showing a high degree of flexibility as well [6]. The N-terminal sequence of the A-chain up to residue Glu1c is characterized by weak electron density and appears to have a high degree of conformational flexibility. This suggests that this sequence does not interact strongly with other parts of the thrombin molecule and may not contribute significantly to the overall structural stability of the enzyme. However, there have been no studies conducted to exclude other biological functions.

Prethrombin-2 is one of two prothrombin activation intermediates and the immediate zymogen precursor to thrombin [4]. Prethrombin-2 is comprised of the B-chain and the full 49-residue A-chain. Mutation of the catalytic triad serine (Ser195) to alanine results in a thrombin that cannot autoproteolyse the A13 peptide from the N-terminus of the A-chain [8]. X-ray structures of Ser195Ala thrombin reveal additional hydrogen bonding between the first 13 residues of the A-chain, the mature A-chain, and the B-chain (Figure 2) [8]. The covalent interaction between the A and B-chains is a disulfide bridge through Cys1(93)-Cys122. The A-chain is stabilized by the Asp1a(92)-Lys9(301) and Arg14d(310)-Glu13(314) ion pairs and the ion quartet Arg4(96)-Glu8(300)-Asp14(306)-Glu14c(309). The ionic interactions include Glu8(300)-Lys202-Glu14c(309), Asp14(306)-Arg137, Lys14a(307)-Glu23, and Glu14e(311)-Lys135-Asn159-Tyr194a. Hydrophobic stacking interactions include Tyr14j(316)-Pro204, and Phe1m(280)-Phe1l(281)-Phe1g(286) in the A13 peptide of the A-chain.

## 4. Evolution

### 4.1. Prothrombin Evolution

Genetic analysis of the clotting factor genes demonstrates that the clotting proteases of the chymotrypsinogen superfamily have evolved as a result of several gene duplications, exon shuffling, and intron sliding events. Gene organization studies reveal that factors VII, IX, and X are closely related and have evolved separately from the homologous genes for factor XII, tissue-type plasminogen activator, and urokinase. Prothrombin has a unique exon organization and is thought to be the ancestral gene in this clotting factor family [9]. 

Analysis of prothrombin genes across seven vertebrate species has established that the most conserved regions of prothrombin are the propeptide region, Gla domain, and the thrombin B-chain. The least conserved regions are the A-chain region and the interconnecting regions between the Gla and kringle domains [9]. There is 54% amino acid identity for the B-chains of thrombin encoding the protease domain while only 40% amino acid identity for the A-chain residues [10]. When conservative substitutions were accounted for, the thrombin B-chains were found to have 75% identity across nine vertebrate species while the A-chain was found to have 42% sequence identity [11]. Sequence alignment of the thrombin A-chain across 11 vertebrate species is shown in Table 1 [12]. Of the ten thrombin A-chain residues that interact with the B-chain, five residues are completely conserved, three are partially conserved, and Ser288 and Thr308 are not conserved (Table 1).

### 4.2. Serine Protease Clan PA Subfamily S1

The subfamily S1A of clan PA encompasses serine proteases bearing the chymotrypsinogen fold and includes proteases with diverse extracellular functions from digestion (e.g., chymotrypsin, trypsin, and proelastase) to complement-mediated immunity (factor B, factor D, factor I, C1r/s, and C2), fibrinolysis (urokinase, tissue-type plasminogen activator, plasminogen, kallikreins) to coagulation (factors VII, IX, X, XI, prothrombin, PC, and PS), and apoptosis (granzymes) to bone remodelling (osteocalcin) [13]. A detailed review of the serine peptidase family and clan classification system is provided in [13]. 

 While some homologous proteases such as trypsin lack an A-chain altogether, other homologous clotting proteins that remain tethered to phospholipid membranes, such as factors Xa, IXa, APC, and VIIa retain the remainder of the zymogen through a covalent disulfide bridge with Cys122 on the B domain [14]. While it is not currently known whether associated zymogen protein domains influence the protease domain in any S1 peptidase, it is considered a plausible scenario [14]. When present in other serine proteases including urokinase and tissue plasminogen activator, a noncatalytic A-chain disulphide bonded to the active B-chain appears to have an allosteric effect on the enzymatic activity [15]. The noncatalytic light chains of plasmin and factor XI also contain binding sites for physiological substrates [16, 17]. Six residues of the prothrombin A-chain region are homologous to the propeptide of chymotrypsinogen. However, the chymotrypsinogen propeptide is not involved in substrate or inhibitor binding [6]. A recent NMR study has also revealed that in the absence of stabilizing ligands, the protease domain regions of highest stability are flanked by the light chain of thrombin. The authors suggest that the A-chain may play a ligand-like role to stabilize and maintain the integrity of the protease domain in the absence of other ligands [18].

### 4.3. Snake Venom Thrombin-Like Enzymes (SVTLEs)

The venoms of many snake genera contain serine proteases that share approximately 26–33% sequence identity with thrombin [19]. One class of these venom proteases is known as thrombin-like due to their ability to cleave fibrinogen to release fibrinopeptide A and/or B [20]. Other thrombin-like activities are found in snake-derived proteases such as cerastobin, batroxobin, ancrod, crotalase, Russel's Viper Venom-V, and thrombocytin, which activate platelets, fibrinogen, factor V, factor XIII, and protein C [20]. SVTLEs and thrombin share a similar catalytic mechanism and have a conserved structure thought to have evolved from a common ancestral protease [21]. As compared to thrombin's three-intrachain and one-interchain (A-B) disulfide bridge, SVTLEs contain twelve cysteine residues, ten of which form disulfide bridges based on homology with trypsin [22]. The remaining two cysteines form a unique and highly conserved disulfide bond in the C-terminal tail of SVTLEs. SVTLEs are either one- or two-chain proteins synthesized as zymogens with proposed activation peptides of six amino acid residues (Q-K-S-S-E-L) [23]. However, none appear to have a light chain that bears homology to thrombin [19]. Additionally, the conserved Cys122 (chymotrypsin numbering) which forms the A-B interchain disulfide of thrombin has been identified as a serine residue in all known SVTLEs [19]. Previous studies have suggested that the amino acid sequences of venom gland serine proteases have diversified in an accelerated manner and that the SVTLE subclass belongs to the most primordial phylogenetic lineage of serine proteases [19]. It is possible that these enzymes diverged from the chymotrypsinogen family prior to the emergence of the A-chain.

## 5. Naturally Occurring Mutations in Human Prothrombin

Of the 50 inherited prothrombin deficiencies reported in the literature, only seven involve mutations in the A-chain. Documented missense mutations of human thrombin include in-frame deletions of three nucleotides in exon 9 (7484/7489 del. GAA), resulting in omission of one of two lysine residues (Lys301 or Lys302) [24, 25]. The deletion of either Lys301 or Lys302 leads to the removal of a salt bridge interaction between Lys301 and Asp292, which is thought to stabilize the centre of the A-chain. The authors suggested that hypoprothrombinemia is caused by the incomplete folding of the A-chain, which is then unable to stabilize the B-chain structure. Two recent studies on the Lys301 deletion prothrombin mutant have suggested that A-chain structure affects the conformation and catalytic properties of thrombin through long range allosteric effects on the active site and insertion loops [25, 26]. Interestingly, a recent crystal structure of thrombin complexed with sulfo-hirudin found that Lys301 participates in a divalent metal binding site between the heavy and light chains of thrombin [27]. The authors hypothesized that this metal binding site contributes to the stabilization of the light chain conformation and may modulate thrombin activity, offering an alternative biochemical explanation for the hemorrhagic diathesis seen in patients with the Lys301 deletion prothrombin mutation [26]. 

Another patient was identified as a compound heterozygote for two prothrombin A-chain missense mutations also located in exon 9 (Glu300Lys and Glu309Lys) [28]. These mutations were designated prothrombin Denver I and II, respectively, and both of these Glu residues are conserved throughout vertebrate species from fish to humans (Table 1). The authors hypothesized that these mutations interfere with the autocatalytic cleavage of the A-chain or activation by factor Xa; however, no confirmatory activity studies have yet been reported. 

Prothrombin San Antonio is a missense mutation resulting in an A to G substitution at nucleotide 7543, resulting in an Arg to His substitution at residue 320. The Arg320-Ile321 bond is one of two prothrombinase-mediated cleavage sites that generate thrombin. Replacement of Arg with His at this site prevents cleavage by factor Xa, forming a dysfunctional molecule [29]. Similarly, Akhavan et al. [30] discovered an A-chain missense mutation resulting from a G to A transition at nucleotide 7312, resulting in the substitution of Arg271 for His. In plasma prothrombin, the Arg271-Thr272 bond is cleaved by factor Xa to form thrombin. The alternative Arg271 replacement by His prevents cleavage by factor Xa at this site and results in the formation of meizothrombin, which has normal amidolytic activity but little fibrinogen clotting activity compared with normal human *α*-thrombin [31]. This observation supports a role for the fully formed A-chain in substrate recognition.

Akhavan et al. [32] also discovered prothrombin Segovia, a G-A mutation at nucleotide 7539 of exon 9 of the prothrombin gene. This resulted in the substitution of Gly319 by Arg. The authors proposed that the substitution, which occurs near the site of factor Xa-mediated cleavage of prothrombin (Arg320-Ile321), altered the conformation of the protein making the cleavage site inaccessible to factor Xa. Taken together, mutational analysis of these naturally occurring A-chain variants suggests that the A-chain of thrombin plays a functional role or roles *in vivo*.

## 6. Thrombin A-Chain Biochemistry

Initial modeling and structural studies of thrombin suggested that the A-chain may contribute to the determination of substrate specificity based largely on its proximity to the active site [33–35]. Early work on plasma-derived bovine thrombin, however, suggested that while the A-chain is strongly associated with the B-chain through an array of noncovalent interactions and a disulphide bond, it does not appear to play a substantial role in the catalytic activity of thrombin [36]. Disulphide reduction, removal of the A-chain, and subsequent refolding of the B-chain demonstrated that the A-chain is not required for proper B-chain folding and that intrachain interactions of the B-chain are sufficient to yield an active enzyme upon refolding. This refolded B-chain cleaved fibrinogen, tosyl-L-arginine methyl ester, and other small peptide substrates at similar rates as refolded two-chain thrombin. These results lead the authors to conclude that the A-chain does not significantly contribute to either the catalytic activity or substrate specificity of thrombin. A similar study by Pirkle et al. [37] confirmed that isolated bovine thrombin B-chain cleaves fibrinogen and is also able to activate factor XIII as shown by the generation of cross-linked fibrin polymers during clotting assays. These studies concluded that prothrombin activation may require the A-chain; however, once mature thrombin is generated, the A-chain is superfluous. This suggests that the A-chain is an activation peptide only. 

Removal of the A-chain of human thrombin by disulphide bond reduction has also been attempted [38]. Reductive unfolding resulted in sequential generation of two partially reduced intermediates prior to conversion to the fully reduced form. Partial loss of B-chain disulphide bonds prior to reduction of the interchain disulphide link was observed, leading to denaturation of the B-chain structure and loss of activity. From this study, the authors concluded that the A-chain may be required for structural stability of the mature thrombin protein through noncovalent interactions, and that the A-chain is required for normal functioning of the active site of thrombin. The authors also conducted disulphide scrambling experiments, demonstrating that thrombin is one of the few proteins that contain disulphide bonds formed between cysteines that occur consecutively in the primary structure of the protein. This arrangement more typically resembles extensively scrambled protein isomers and was found to result in weak conformational stability as the native protein is easily converted to disulphide scrambled isomers at low denaturant concentrations [38]. The authors proposed that this unique disulphide arrangement and consequent conformational flexibility may be crucial for the multiple bioregulatory functions of thrombin. 

A number of linkage studies have investigated the effects of salt, pH, and temperature on thrombin amidase activity, highlighting not only the importance of both cation and anion binding sites, but also the allosteric communication between these sites and the active site of thrombin [39–46]. In particular, substrate binding to the catalytic pocket was found to be controlled by two ionizable groups while the catalytic activity of thrombin was controlled by a more complex linkage scheme involving three ionizable groups [46]. The authors postulated that the two ionizable groups that affect both *k*_cat_ and *k*_*m*_ were the active site histidine and the B-chain amino terminus, as these residues play similar roles in other serine proteases. The third ionizable group, which affects catalytic activity but not substrate binding, was suggested to be part of the anion binding exosite of thrombin [44, 46]. While these early studies did not specifically identify the A-chain as an allosteric effector of thrombin activity, they did emphasize the biological importance of allostery in thrombin function and served as the foundation for future mutagenesis studies addressing potential long-range linkages between the thrombin active site and the A-chain.

## 7. Mutagenesis Studies

Prothrombin and prethrombin-2 mutagenesis and recombinant expression studies of A-chain residues have revealed that at least some A-chain residues impart functional effects on thrombin activity. In an extensive mutagenesis study, Tsiang et al. [47] functionally mapped surface exposed residues of thrombin that were capable of participating in H-bonds and electrostatic interactions. Charged and polar residues were chosen for mutation as these were considered most likely to participate in the binding of charged ligands. Prothrombin A-chain residues Ser288, Glu290, Asp292, Lys301, Lys302, Ser303, Lys307, Arg310, Glu311, Glu314, and Asp318 were individually mutated to alanine, expressed in COS7 cells, and the unpurified mutants were functionally analyzed for amidolytic activity, fibrinogen clotting, protein C activation, and inhibition by thrombin aptamer. A triple mutant (Ser288Ala/Glu290Ala/Asp292Ala) had significantly enhanced amidolytic and fibrinogen clotting activity while a double mutant (Glu314Ala/Asp318Ala) had reduced fibrinogen clotting activity, but slightly enhanced protein C activation. Conversely, the Lys307Ala mutant possessed significantly reduced amidolytic and clotting activity, as well as reduced protein C activation. These results demonstrate that charged A-chain residues contribute in thrombin activity toward both pro- and anticoagulant substrates.

More recently, a site-directed mutagenesis study was performed on charged A-chain residues known to be involved in inter- and intramolecular interactions [48]. Prethrombin-1 A-chain mutants were expressed in BHK cells and purified prior to functional analysis. Of the analyzed mutants, Glu300, Asp306, Glu309, and Arg296 were each found to be functionally compromised in terms of hydrolysis of the chromogenic substrate H-D-Phe-Pro-Arg-p-nitroanilide (FPR), release of fibrinopeptides A and B, activation of protein C, and Protease Activated Receptor-1 cleavage (PAR1). In particular, the Arg296Ala mutant was most severely compromised with a 65-fold reduction in FPR hydrolysis. The concomitant reduction in cleavage of fibrinogen, PAR1, and protein C confirmed the molecular origin of active site perturbation more than 20 angstroms from the active site. The Arg296 side chain is stabilized by strong ionic contacts in the ion quartet Arg296-Glu300-Asp306-Glu309 and also interacts with Trp29 and Trp207 in the B-chain via van der Waals forces. Alanine substitution of Arg296 abrogates the ionic interaction, weakening the ion quartet and perturbation of the A-chain stability through Arg296Ala substitution and is thought to propagate long-range to the active site via Trp29 and Trp207. 

Papconstantinou et al. [48] also used alanine scanning mutagenesis to investigate the functional effects of the naturally occurring mutations prothrombins Denver I and II (Glu300Lys and Glu309Lys), Segovia (Gly319Arg), and San Antonio (Arg320His). Arg320His and Gly319Arg affect the factor Xa P1 and P2 recognition sites, respectively, and these mutations have previously been attributed to perturbation of the zymogen activation process. The bleeding diatheses associated with Glu300Lys and Glu309Lys may result from their interference with the autocatalytic cleavage of the A-chain or factor Xa-mediated activation [28]. However, considering that these two mutations occur in the central region of the A-chain, it seems plausible that these mutations disrupt the structural stabilization of the A- and B-chains after thrombin activation. Alanine substitution of Glu300 and Glu301 was found to result in a reduction in fibrinogen cleavage and PAR1 activation. It is expected that the charge reversal of the naturally occurring Glu300Lys and Glu309Lys mutations is substantially more disruptive than the alanine substitution conducted. 

The naturally occurring prothrombin Lys301 deletion has been most extensively studied of all A-chain mutations. This mutation has been found to result in reduced substrate turnover for Phe-Pip-Arg-p-nitroaniline, reduced antithrombin-III and protein C interaction, less robust platelet activation, and reduced sodium ion sensitivity. By contrast, thrombin-thrombomodulin and thrombin-platelet glycoprotein Ib*α* interactions were unaffected [25]. Further structural characterization revealed decreased stability of the Lys301 deletion mutant and a weakening in the A-B interchain interactions, resulting in faster dissociation of the A-chain upon disulfide scrambling [26]. Molecular dynamics simulation of the effects of Lys301 deletion revealed geometric distortion of the catalytic triad and alteration to the aryl-binding site within the active site, resulting in substrate restriction to the active site cleft. In particular, there were changes to the S2 subsite (Trp60d loop) and transition of Trp215 (S3 subsite). These changes collectively resulted in reduced catalytic activity, confirming that alterations in A-chain residues have the potential to transmit long-range allosteric effects to the active site [26].

## 8. Signaling and Disease

Thrombin plays a role in many processes linked to cancer including thrombosis, inflammation, and tissue repair and remodeling (reviewed in [49]). Recent work by Ebert et al. [50] has identified the thrombin A-chain as a potential diagnostic tool for gastric cancer. Patients with dyspeptic symptoms could be distinguished from those with gastric cancer by comparing levels of circulating liberated A-chain with both specificity and sensitivity of this marker found to be 80%. The authors speculated that the decreased circulating A-chain levels observed in cancer patient serum samples correspond to higher concentrations of intact active thrombin in the tumor microenvironment possibly due to reduced local proteolytic degradation of thrombin. While this does not imply a specific role for the thrombin A-chain in the tumor environment, it does suggest that additional proteolytic events occur to liberate the A-chain from the localized thrombin molecule. Given that activation peptides released from prothrombin and other coagulation proteins have signaling effects [51–53], it is possible that released A-chain peptides may have a similar function. A recent study investigated the ability of various thrombin fragments to act as host defense peptides [54]. While no antimicrobial or anti-inflammatory capabilities were detected for A-chain peptides examined under physiological conditions in this study, future research involving (pro) thrombin fragmentation by a wider array of host, bacterial, and viral proteases may identify A-chain peptides that function within the immune system, further expanding the biological roles of thrombin.

## 9. Summary

In this paper, we have summarized the current literature on the thrombin A-chain. While thrombin is well known to be an allosterically-regulated protease, there is a relative paucity of data addressing the contribution of the A-chain to thrombin function. Early studies on bovine thrombin concluded that the B-chain alone is an active serine protease, suggesting the A-chain is an activation peptide only. However, subsequent mutagenesis studies with naturally occurring A-chain mutations indicate that this polypeptide chain is of structural and/or functional importance. Given the wide array of substrates and cofactors with which thrombin interacts, the extent of the physiologic and pathologic roles of the A-chain remains unclear. Mutational analysis and structural studies offer the most promising approach to clarify the nature of A-chain function. Our laboratory is focusing on the mutation of conserved thrombin A-chain residues to analyze functional effects. We also intend to further characterize impaired mutants with crystallography studies to clarify the physiological role of the thrombin A-chain as an allosteric effector and not simply an activation remnant.

## Figures and Tables

**Table 1:** Prothrombin A-chain sequence alignment across 11 vertebrate species. NCBI CDD Pfam09396 [12]. In the third column, conserved residues are shown in regular font, and nonconserved residues are marked in italic font. 12 human prothrombin A-chain residues interact with the B-chain and are shown underlined. Human prothrombin numbering is provided underneath the sequences.

Species	Common name	A-chain sequence
Homo sapiens	Human	T*ATS*EYQ*T*FFNPRTFG*S*GEADCG*L*RPLFEKKSL*E*DK*T*ERELLESY*I*DGR
Mus musculus	Mouse	T*TDA*EFH*T*FFNEKTFG*L*GEADCG*L*RPLFEKKSL*K*DT*T*EKELLDSY*I*DGR
Rattus norvegicus	Norway rat	T*TDA*EFH*T*FFDERTFG*L*GEADCG*L*RPLFEKKSL*T*DK*T*EKELLDSY*I*DGR
Bos Taurus	Cow	T*SED*HFQ*P*FFNEKTFG*A*GEADCG*L*RPLFEKKQV*Q*DQ*T*EKELFESY*I*EGR
Elaphe sp.	Elephant	T*TIQ*QHE*T*FFDPKTFG*E*GEADCG*I*RPLFEKKKI*S*DS*T*ENELLESY*L*QGR
Gallus gallus	Chicken	T*IFQ*EFK*T*FFDEKTFG*E*GEADCG*T*RPLFEKKQI*T*DQ*S*EKELMDSY*M*GGR
Struthio camelus	Ostrich	T*VLQ*EYK*T*FFDDKTFG*S*GEADCG*I*RPLFEKKKI*K*DK*S*EKELLESY*I*GSR
Crocodylus niloticus	Nile crocodile	T*SIP*EYK*V*FFDPKTFG*S*GEADCG*I*RPLFEKKNI*A*DK*T*EKDLLESY*I*EGR
Danio rerio	Zebra fish	T*TLD*QRK*A*FFNPRSFG*N*GELDCG*E*RPLFEKINK*A*DK*N*EKELLMSY*T*GSR
Oncorhynchus mykiss	Rainbow trout	T*LSG*PRQ*S*FFSPQSFG*S*GELVCR*E*RPMFEKMSK*K*DG*R*EQELIDSY*Q*GGR
Tetraodon nigroviridis	Puffer fish	T*TLS*SRK*Q*FFNPRTFG*Q*GENDCG*Q*RPLFEKISK*K*DA*K*EDELLESY*R*EKR
Xenopus laevis	African clawed frog	T*TTE*EHQ*T*FFDEKTFG*S*GEAVCG*L*RPLFEQKSV*E*DK*G*EKELMESY*M*QGR
Human prothrombin numbering:		272 282 292 302 312
